# Regional dopaminergic dysfunction patterns discriminate Parkinson’s disease from multiple system atrophy parkinsonian subtype

**DOI:** 10.1016/j.prdoa.2026.100451

**Published:** 2026-05-23

**Authors:** Dan Xu, Chenhao Jia, Tianhao Zhang, Chao Ren, Wenjia Zhu, Han Wang, Li Huo

**Affiliations:** Peking Union Medical College Hospital, China

**Keywords:** Parkinson’s disease, Multiple system atrophy parkinsonian subtype, ^18^F-DOPA PET, Caudate nucleus, cerebellum

## Abstract

**Background:**

Parkinson’s disease (PD) and multiple system atrophy parkinsonian subtype (MSA-P) are clinically similar α-synucleinopathies, making differential diagnosis challenging. This study aimed to identify distinct regional dopaminergic dysfunction patterns using quantitative ^18^F-DOPA PET to differentiate the two disorders.

**Methods:**

We prospectively recruited 20 PD patients, 12 MSA-P patients, and 9 healthy controls. All underwent ^18^F-DOPA PET imaging. Images were normalized using an adaptive probabilistic brain atlas. Voxel-wise and region-of-interest (ROI) analyses were performed with Statistical Parametric Mapping 12, controlling for age, sex, and disease duration. ROI comparisons used ANCOVA with Bonferroni post-hoc tests.

**Results:**

Both patient groups showed severe putaminal dopaminergic loss compared to controls. A key differentiator was caudate nucleus involvement: SUVR was significantly lower in MSA-P than in PD (*P* = 0.031) and controls (*P* = 0.006), while PD did not differ from controls. Cerebellar SUVR was also significantly reduced in MSA-P compared to PD (*P* = 0.026) and controls (*P* = 0.007), despite no clinical cerebellar signs in MSA-P patients. Putamen uptake did not differentiate PD from MSA-P. Voxel-wise analysis confirmed reduced uptake in bilateral caudate, posterior cerebellar lobes, and vermis in MSA-P vs PD. No significant correlations between SUVR and clinical severity were found after correction for multiple comparisons.

**Conclusions:**

^18^F-DOPA PET reveals distinct topographic patterns: PD shows predominant putaminal impairment with caudate sparing, whereas MSA-P is characterized by concurrent caudate and cerebellar dopaminergic dysfunction. Caudate and cerebellar ^18^F-DOPA uptake are potential imaging biomarkers for differentiating MSA-P from PD.

## Highlights

• Adaptive probabilistic atlas enables MRI-free ^18^F-DOPA PET spatial normalization.
• Caudate nucleus uptake reduction specific to MSA-P compared to PD.
• Cerebellar dopaminergic dysfunction in MSA-P without clinical signs.
• Caudate and cerebellar ^18^F-DOPA uptake differentiate MSA-P from PD.

## 1 Introduction

Both Parkinson's disease (PD) and multiple system atrophy-parkinsonian subtype (MSA-P) are α-synucleinopathies characterized by considerable clinical overlap in their motor manifestations, notably bradykinesia, muscular rigidity, and impaired postural stability [1], [2]. This clinical overlap often leads to diagnostic challenges, with approximately 20% of MSA-P patients misdiagnosed as having PD, even at specialized centers [3], [4]. While PD is characterized predominantly by neuronal Lewy bodies [5], MSA-P is defined by glial cytoplasmic inclusions [6], pathologies that drive distinct topographical progression patterns. Although the distinct pathological hallmarks of PD and MSA-P are well established (Lewy bodies vs. glial cytoplasmic inclusions), the mechanisms that drive selective vulnerability of specific dopaminergic pathways remain incompletely understood [7]. This knowledge gap underscores the need for biomarkers that can accurately differentiate these disorders and elucidate their distinct pathophysiological substrates.

^18^F-fluorodihydroxyphenylalanine (^18^F-DOPA) PET imaging provides a non-invasive method to quantify presynaptic dopaminergic integrity through measurement of aromatic L-amino acid decarboxylase (AADC) activity [8]. Prior studies demonstrate regional ^18^F-DOPA uptake reductions in both PD and MSA-P [9], [10]. However, significant controversies persist regarding its diagnostic specificity in differentiating PD and MSA-P [9]. The discriminative value of extra-striatal uptake patterns of parkinsonism remains inadequately validated [8], [9]. These limitations emphasize the need for rigorous quantification of regional dopaminergic dysfunction to establish robust imaging biomarkers.

To address this need, we conducted a prospective cohort study using quantitative ^18^F-DOPA PET imaging in PD and MSA-P patients. This methodology leverages the technique's high spatial resolution and pathophysiological specificity to map dopamine synthesis capacity across striatal and infratentorial regions. We aimed to identify differential dopaminergic impairment patterns between PD and MSA-P while advancing objective diagnostic criteria for clinical application.

## 2 Methods

### 2.1 Participants

We prospectively and sequentially recruited patients with PD and MSA-P who visited the neurology department of Peking Union Medical College Hospital (PUMCH) from September 1, 2018, to August 31, 2022. The diagnosis of PD and MSA-P was based on widely accepted criteria [1], [2]. No MSA-P patients had clinical cerebellar signs. Nine controls without nigrostriatal system defects (including essential tremors, healthy individuals, drug-induced parkinsonism, and dystonia) were also recruited. Individuals with a history of other central nervous system diseases, including other neurodegenerative disorders such as progressive supranuclear palsy or frontotemporal lobar degenerations, were excluded. We prospectively documented all participants’ clinical and demographic profiles, including age, sex, disease duration, Hoehn-Yahr (H-Y) stage, and Unified Parkinson’s Disease Rating Scale Part III (UPDRS-III) scores. Furthermore, no cognitive impairments were detected in any participant during neurological physical examination. Cranial CT or MRI (obtained at our hospital or externally) revealed no significant atrophy of the frontal, temporal, or midbrain regions in the included patients. This study was approved by the Institutional Review Board of PUMCH (Clinical trial number JS-2864). Written informed consent was signed before study participation.

### 2.2 Radiosynthesis and imaging acquisition

The synthesis of ^18^F-DOPA began with the reaction of 6-nitroveratraldehyde and dried ^18^F fluoride. This produced 2-[18F] fluoro-4,5-dimethoxybenzaldehyde. The product was purified using a tC18 cartridge. Next, the aldehyde group was reduced to a primary alcohol using NaBH4. The resulting hydroxyl group was then iodinated with concentrated HI. After purification, the iodinated compound was reacted with a Schiff’s base in the presence of a phase-transfer catalyst. This alkylation step yielded protected ^18^F-DOPA. The protecting groups were removed by heating with concentrated HI at 160°C. Finally, the crude product was purified by HPLC and formulated for injection.

Anti-Parkinsonian medications and other drugs potentially affecting ^18^F-DOPA uptake (e.g., diazoxide or cortisone) were discontinued 24 h before the study.

Participants were administered a single intravenous bolus of ^18^F-DOPA (370–550 MBq). A low‑dose CT scan was obtained at 90 min after injection, followed by a 20‑minute PET scan. The brain PET/CT scan was carried out on a time‑of‑flight PET/CT scanner (Polestar m660, Sinounion, China). Images were reconstructed using an ordered subsets expectation maximization algorithm (2 iterations, 10 subsets, 192 × 192 matrix) and corrected for CT‑based attenuation, dead time, random events, and scatter.

### 2.3 ^18^F-DOPA PET imaging analysis

We implemented an MRI-free method because some participants did not undergo complete brain MRI. All PET images underwent spatial normalization into the Montreal Neurological Institute (MNI) space via an MRI-free algorithm that employs an adaptive probabilistic brain atlas [11]. Following this, spatial smoothing was applied with an 8-mm full-width at half maximum (FWHM) Gaussian kernel. For quantitative analysis, the standardized uptake value ratio (SUVR) for each voxel in the 18F-FDOPA PET images was derived by normalizing the uptake against the occipital lobe as the reference region [12]. Based on previous ^18^F-DOPA PET studies [13], [14], we defined a set of regions of interest (ROIs) for subsequent analyses. For voxel-wise comparisons, an explicit binary mask encompassing six a priori selected regions was applied to limit the search volume: caudate nucleus, putamen, globus pallidus, thalamus, substantia nigra, and cerebellum. For ROI analysis, mean SUVR values were extracted from a subset of four regions (caudate nucleus, putamen, cerebellum, and substantia nigra pars compacta) using the Wake Forest University (WFU)_PickAtlas [15] in MNI space. The specific AAL3 atlas regions merged to define each ROI are provided in Supplementary Table 1.

### 2.4 Statistical analysis

Descriptive statistics were expressed as mean ± standard deviation (SD) for continuous variables (age, disease duration, UPDRS-III scores, and regional SUVR values) and as frequencies with percentages for categorical variables (sex). Between-group comparisons for continuous variables were performed using one-way analysis of variance (ANOVA) followed by post-hoc tests, while the chi-square test was used for sex distribution.

Voxel-wise analysis was performed using Statistical Parametric Mapping 12 (SPM12). For voxel-wise analysis, we focused on the direct comparison between MSA-P and PD patients. A two-sample *t*-test was performed with sex, age, and disease duration as covariates. Voxel-wise statistical significance was set at a threshold of P < 0.05 after false discovery rate (FDR) correction for multiple comparisons, with a cluster extent threshold of > 500 voxels.

For ROI-wise comparisons among the three groups (PD, MSA-P, and controls), a univariate general linear model analysis of covariance (ANCOVA) was used, with age and sex as covariates. For direct comparisons between PD and MSA-P, age, sex, and disease duration were included as covariates. Post-hoc pairwise comparisons were performed using Bonferroni correction. Partial eta squared (η^2^) was reported as a measure of effect size.

To assess whether regional ^18^F-DOPA uptake reflects disease severity, Spearman‘s rank correlation coefficients were calculated between SUVR values of the four regions and clinical metrics (UPDRS-III, H-Y stage, disease duration) in PD and MSA-P patients separately. A Bonferroni-corrected significance threshold of P < 0.0042 (0.05/12) was applied to account for multiple comparisons (four regions × three clinical metrics). All statistical analyses were performed using SPSS version 22.

## 3 Results

### 3.1 Clinical data of the subjects

Twenty PD and 12 MSA-P patients were recruited. While a statistically significant difference in age was observed across the three groups, post-hoc analysis revealed that the PD group was significantly younger than the control group (*P* = 0.013), while MSA-P patients (59.6 ± 4.1 years) showed no significant age difference versus PD (*P* = 0.433) or controls (*P* = 0.092), despite being numerically younger than controls. The three groups were well-matched for sex distribution. Disease duration was numerically longer in PD (6.00 ± 4.89 years) versus MSA-P (3.50 ± 1.68 years), though this difference did not reach statistical significance (*P* = 0.099). Notably, UPDRS-III data were available for 17 of the 20 PD participants and 10 of 12 MSA-P patients. Motor severity assessments revealed no significant differences in Hoehn & Yahr stage (*P* = 0.638) or UPDRS-III scores (PD: 30.18 ± 15.05 [n = 17/20]; MSA-P: 34.20 ± 11.63 [n = 10], *P* = 0.475) (Table 1).

**Table 1:** Baseline demographic and clinical profile of PD, MSA-P and controls.

Group	PD	MSA-P	Controls	*P* value
Number	20	12	9	
Ages (years)*	57.1 ± 10.9	59.6 ± 4.1	66.1 ± 6.5	0.043
Sex (number)				
Male	10	7	3	0.520
Female	10	5	6	
Disease duration	6.00 ± 4.89	3.5 ± 1.68	−	0.099
H-Y stage	2.00 ± 0.56	2.00 ± 0.29	−	0.638
UPDRS-III score (number)	30.18 ± 15.05(17)	34.20 ± 11.63(10)	−	0.475

* Age differed significantly across groups (*P* = 0.043, ANOVA). Post-hoc tests revealed: MSA-P vs. PD: *P* = 0.433, MSA-P vs. Control: *P* = 0.092, PD vs. Control: *P* = 0.013.

Abbreviations: H-Y stage: Hoehn-Yahr stage; UPDRS-III score: Unified Parkinson's Disease Rating Scale Part III score.

### 3.2 Voxel-wise analysis

**Fig. 1:**
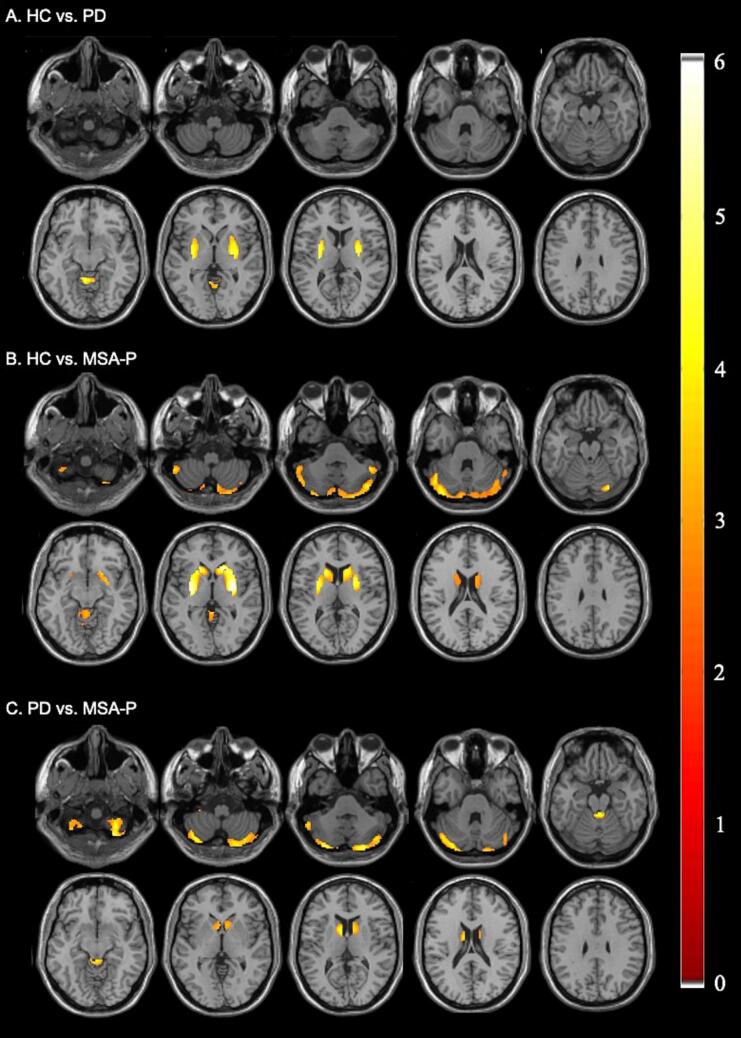
Voxel-wise comparisons of ^18^F-DOPA PET (FDR-corrected P < 0.05, cluster extent > 500 voxels). Yellow = higher uptake in former group. (A) HC vs. PD: HC shows higher putamen uptake than PD. (B) HC vs. MSA-P: HC shows higher caudate, putamen and cerebellum uptake than MSA-P. (C) PD vs. MSA-P: PD shows higher caudate, cerebellar, and vermal uptake than MSA-P. (For interpretation of the references to colour in this figure legend, the reader is referred to the web version of this article.)

Voxel-wise comparisons were performed between each pair of groups (Fig. 1). Compared to controls, PD patients showed significantly reduced ^18^F-DOPA uptake in the bilateral putamen (Fig. 1A). MSA-P patients showed more extensive reductions compared to controls, involving the bilateral caudate nuclei, putamen and cerebellum (Fig. 1B). Direct comparison between MSA-P and PD patients revealed that MSA-P patients had significantly lower uptake in the bilateral caudate heads, bilateral posterior cerebellar lobes, and cerebellar vermis compared to PD patients (Fig. 1C). Detailed cluster information for the MSA-P vs. PD comparison is provided in Supplementary Table 2.

### 3.3 Representative images

**Fig. 2:**
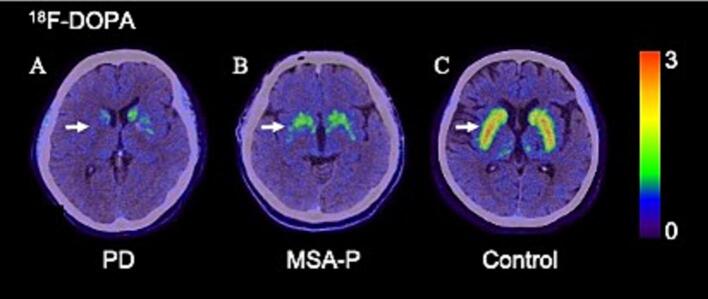
**Representative ^18^F-DOPA PET images of a healthy control, a PD patient, and a MSA-P patient.** (A) PD patient (female, 65 years, disease duration 13 years): asymmetrically reduced DOPA uptake in bilateral basal ganglia (white arrow). (B) MSA-P patient (male, 55 years, disease duration 1.5 years): symmetrically reduced bilateral basal ganglia uptake (white arrow). (C) Control (male, 77 years, essential tremor): normal bilateral basal ganglia uptake (white arrow).

Representative ^18^F-DOPA PET images of a healthy control, a PD patient, and an MSA-P patient are shown in Fig. 2. The PD patient exhibited asymmetrically reduced uptake in the bilateral basal ganglia with relative sparing of the caudate, while the MSA-P patient showed symmetrically reduced uptake involving both the putamen and caudate. These visual findings are consistent with the quantitative ROI analyses presented below.

### 3.4 ROI‑wise ^18^F-DOPA values

After adjusting for age and sex, significant differences among the three groups were observed in the caudate nucleus, putamen, and cerebellum, but not in the substantia nigra pars compacta (Table 2). Post-hoc comparisons with Bonferroni correction revealed that MSA-P patients had significantly lower caudate SUVR than both healthy controls (*P* = 0.006) and PD patients (*P* = 0.031), whereas PD patients did not differ from controls (*P* = 0.999). For the putamen, both PD and MSA-P patients showed significantly lower SUVR compared to controls (both *P* < 0.001), with no difference between the two patient groups (*P* = 0.718). Cerebellar SUVR was significantly reduced in MSA-P compared to controls (*P* = 0.007) and to PD (*P* = 0.026), while PD and controls did not differ (*P* = 0.999). When directly comparing PD and MSA-P with additional adjustment for disease duration, the differences remained significant for the caudate and cerebellum (Supplementary Table 3).

**Table 2:** Regional ^18^F-DOPA SUVR values among PD, MSA-P, and controls.

Region	Group	Estimated marginal mean ± SE	*F* (df)	*P* value	Post-hoc pairwise comparisons (Bonferroni)
Caudate	Control	1.694 ± 0.051	5.47 (2, 34)	0.008	MSA-P < Control (*P* = 0.006); MSA-P < PD (*P* = 0.031);PD vs Control (P = 0.999)
	PD	1.603 ± 0.040			
	MSA-P	1.358 ± 0.053			
Putamen	Control	2.495 ± 0.116	13.96 (2, 34)	<0.001	Control > PD (*P* < 0.001); Control > MSA-P (*P* < 0.001);PD vs MSA-P (*P* = 0.718)
	PD	1.860 ± 0.092			
	MSA-P	1.740 ± 0.120			
Cerebellum	Control	1.096 ± 0.017	5.48 (2, 34)	0.009	MSA-P < Control (*P* = 0.007); MSA-P < PD (*P* = 0.026);PD vs Control (*P* = 0.999)
	PD	1.056 ± 0.013			
	MSA-P	1.006 ± 0.017			
SN_pc	Control	1.476 ± 0.077	0.88 (2, 34)	0.424	No significant differences
	PD	1.395 ± 0.061			
	MSA-P	1.421 ± 0.079			

Estimated marginal means and statistical comparisons were derived from a univariate general linear model (ANCOVA) with age and sex as covariates. Significant *P* values (<0.05) are bolded.

Abbreviations: MSA-P: Multiple system atrophy-parkinsonian subtype; PD: Parkinson’s disease; SN_pc: Substantia nigra pars compacta; Ave = Average.

### 3.5 Diagnostic performance of regional SUVR

ROC analysis was performed to evaluate the ability of caudate and cerebellar SUVR to distinguish MSA-P from PD. The area under the curve (AUC) for caudate SUVR was 0.796 (95% CI: 0.646–0.933). Using a cut-off value of ≤ 1.524, caudate SUVR differentiated MSA-P from PD with a sensitivity of 83.3% (10/12) and a specificity of 70.0% (14/20). For cerebellar SUVR, the AUC was 0.713 (95% CI: 0.538–0.879). At a cut-off of ≤ 1.021, sensitivity was 66.7% (8/12) and specificity was 75.0% (15/20). These findings suggest that caudate SUVR has moderate diagnostic accuracy for discriminating MSA-P from PD, whereas cerebellar SUVR provides additional but weaker discriminatory value. Detailed ROC results are presented in Supplementary Table 4.

### 3.6 Correlation analyses

In both PD and MSA-P groups, no significant correlations were observed between any regional SUVR and the three clinical measures after correction (all P > 0.0042). Detailed correlation results are presented in Supplementary Table 5.

## 4 Discussion

This study provides novel in vivo evidence of distinct dopaminergic dysfunction patterns in moderate-stage PD and MSA-P patients using quantitative ^18^F-DOPA PET imaging. Our key findings demonstrate: while PD exhibited selective putaminal impairment with relative caudate sparing, MSA-P showed both a characteristic concurrent reduction in dopa uptake in the caudate nucleus and cerebellum despite absent cerebellar signs, with significantly greater caudate involvement versus PD. These differential topographic patterns advance our understanding of α-synucleinopathy trajectories and offer practical diagnostic biomarkers.

Fundamentally, ^18^F-DOPA PET measures presynaptic dopaminergic integrity through uptake and decarboxylation by aromatic L-amino acid decarboxylase (AADC), reflecting dopamine synthesis capacity rather than mere perfusion effects [16]. ^18^F-DOPA PET in 1983 first revealed neostriatal neurotransmitter precursor distribution and pharmacologic responsiveness in living monkeys [17]. While ^18^F-DOPA PET effectively distinguishes healthy subjects from parkinsonism patients [18], [19], their value in differentiating PD from MSA-P remains controversial.

Consistent with the ROI findings, voxel-wise analysis confirmed that MSA-P patients had significantly reduced ^18^F-DOPA uptake in the bilateral caudate nucleus, posterior cerebellar lobes, and cerebellar vermis compared to PD patients (Fig. 1). This topographical pattern supports the notion that MSA-P involves more widespread dopaminergic dysfunction beyond the putamen, affecting both the caudate and infratentorial structures.

Although Xian and colleagues (2021) noted significantly diminished ^18^F-DOPA uptake in the bilateral striatum (putamen and caudate) of both PD and MSA-P patients compared to healthy controls, their analysis failed to reveal a significant difference in the extent of this reduction between the two patient cohorts [20]. Goldstein et al. (2008) conversely demonstrated reduced putamen-to-caudate DOPA uptake ratios in PD versus MSA-P (p < 0.01), reflecting distinct topographical patterns of dopaminergic degeneration [21]. Supporting our findings, earlier research has also documented a pattern of relatively spared caudate nucleus uptake in PD, in contrast to the more uniform striatal involvement seen in MSA [22]. Employing both voxel-wise and ROI analyses, our results demonstrated significantly reduced ^18^F-DOPA uptake in the putamen for both PD and MSA-P patients compared to HC, with no significant difference observed between the PD and MSA-P groups for this region. In contrast, caudate nucleus uptake in MSA-P patients showed significantly lower than both PD patients and controls, while no significant difference was revealed between the latter two groups.

The distinct pattern of dopaminergic decline in the basal ganglia, characterized by relatively preserved caudate function in PD in contrast to its impairment in MSA-P, together with comparable putaminal depletion in both disorders, supports the concept of divergent pathophysiological involvement [21], [22] and indicates that caudate ^18^F-DOPA PET uptake may serve as a useful imaging biomarker for differentiating PD from MSA-P. Postmortem anatomical studies provided mechanistic insights for these DOPA PET findings: preserved caudate dopamine in PD [23] aligns with predominant neurodegeneration in lateral substantia nigra pars compacta putamen-projecting neurons[24], [25], [26]. Conversely, MSA exhibited diffuse nigral degeneration without Lewy bodies [27], causing greater nigrocaudate disruption.

The observation of reduced cerebellar ^18^F-DOPA uptake in our MSA patients is intriguing, given that none of the MSA-P patients demonstrated clinical cerebellar signs and the cerebellum is not a primary dopaminergic terminal region. Physiologically, cerebellar 18F-DOPA signal is extremely low due to scarce expression of aromatic amino acid decarboxylase (AADC) in this area. Moore et al. performed high‑resolution ^18^F-DOPA PET in healthy humans and reported that the striatum exhibits the highest uptake, whereas the cerebellum was not listed among any regions with significant specific binding [28]. Similarly, a systematic review of normal biodistribution confirmed that cerebral ^18^F-DOPA uptake is negligible except in the basal ganglia, and the cerebellum is routinely used as a non‑specific reference region [29]. Therefore, any minor fluctuations near background may be amplified in disease states. Several mechanisms could account for the reduced cerebellar ^18^F-DOPA uptake in our patients, either alone or in combination. First, a functional network (cross‑talk) effect is plausible. Previous fluorodeoxyglucose (FDG)‑PET studies have demonstrated cerebellar hypometabolism in early MSA [30], and recent spatial independent component analysis revealed that the cerebellar metabolic network is significantly correlated with striatal dopamine transporter availability [13], suggesting remote metabolic consequences of basal ganglia dysfunction via cerebello‑striatal circuits. Second, partial volume effects due to cerebellar atrophy, which is common in MSA, can artificially lower measured PET signals by mixing with cerebrospinal fluid; without correction, such effects may cause a 19‑49% underestimation of true tracer uptake [31]. Third, although 18F-DOPA primarily reflects AADC activity in monoaminergic neurons, it can also be taken up by noradrenergic (locus coeruleus) and serotonergic (raphe nuclei) terminals [32]. In the MSA‑cerebellar subtype, vesicular monoamine transporter type 2 (VMAT2) PET has shown reduced cerebellar binding, indicating loss of non‑dopaminergic monoaminergic afferents [33]. However, the contribution of these non‑dopaminergic systems to cerebellar ^18^F-DOPA signal is likely minor, because the cerebellum receives sparse noradrenergic and serotonergic innervation relative to other brain regions. Therefore, the decreased cerebellar ^18^F-DOPA uptake observed in our study most probably reflects a combination of network‑driven metabolic suppression, partial volume effects, and non‑specific background variability, rather than direct dopaminergic denervation of the cerebellum.

No significant correlations were found between regional SUVR and clinical severity after correction. This absence of correlation may be explained by the limited sample size, which reduces power to detect modest associations, and the relatively narrow range of disease severity in our cohort (most patients were at H-Y stage 2). These findings suggest that caudate and cerebellar SUVR are more valuable for differential diagnosis than for monitoring disease progression in small cohorts.

Brain PET image spatial normalization commonly relies on MRI-based methods [34]. To further validate this MRI-free normalization approach in ^18^F-DOPA PET, prior work by Zhang et al. demonstrated its consistency with MRI-based gold-standard methods across three quantitative dimensions: voxel-wise global correlation, clinical metric quantification, and spatial overlap of significant regional differences [11]. This method has demonstrated applicability across various PET tracers and brain disorders, including FDG (1^8^F-fluro-2-deoxyglucose), tau-specific AV-1451 (1^8^F-flortaucipir) and PIB (11C-Pittsburgh Compound B) in PD, Alzheimer’s disease and frontotemporal dementia [11]. Besides, it has also enabled visualization of in vivo Tau distribution patterns in anti-IgLON5 disease using Florzolotau (^18^F) PET imaging [35]. In this study, we uniquely integrated ^18^F-DOPA PET with this atlas-based approach. Results align with prior DOPA PET-CT studies [21], [22]. Consequently, the atlas-based method achieves accurate spatial normalization of ^18^F-DOPA PET images into standard space without requiring structural MRI, even in pathological states. Additionally, it provides a possibility for objective PET analysis when MRI data are unavailable, representing a strength of this study.

Several limitations of this study should be considered. The sample size of the MSA-P group (n = 12) is relatively small, which limits statistical power and may reduce the ability to detect subtle regional differences, particularly within infratentorial structures. This limited sample size also constrains subgroup analyses and may affect the generalizability of our findings to the broader MSA-P population. Consequently, the diagnostic performance indicators derived from ROC analysis (e.g., AUC, cut-off values, sensitivity, specificity) have wide confidence intervals and should be considered exploratory; validation in larger independent cohorts is required before clinical application. Although current clinical criteria were strictly applied, the absence of neuropathological verification or α-synuclein seeding assays (e.g., RT-QuIC) introduces potential misclassification bias, as approximately 20% of clinically diagnosed MSA-P cases may represent PD variants at autopsy. Future studies should prioritize larger, multicenter cohorts with biomarker-confirmed diagnoses and high-resolution PET-MRI co-registration to validate our findings and improve generalizability.

It appears that the system link may have pointed to the wrong section (Limintations instead of Funding). However, we were unable to located any Funding section in the current proof. It was properly included in the originally submitted manuscript. The correct funding statement for our study is as follows:

Funding: this work was financially supported by National Natural Science Foundation of China (No. 82171255); National High Level Hospital Clinical Research Funding (2022-PUMCH-B-018); CAMS Innovation Fund for Medical Sciences (CIFMS) (2021-I2M-C&T-B-009).

## CRediT authorship contribution statement

**Dan Xu:** Writing – original draft, Visualization, Validation, Investigation, Funding acquisition, Formal analysis, Data curation. **Chenhao Jia:** Visualization, Investigation. **Tianhao Zhang:** Methodology, Formal analysis. **Chao Ren:** Investigation. **Wenjia Zhu:** Writing – review & editing, Methodology, Investigation. **Han Wang:** Writing – review & editing, Supervision, Funding acquisition, Conceptualization. **Li Huo:** Supervision, Funding acquisition, Conceptualization.

## Declaration of competing interest

The authors declare that they have no known competing financial interests or personal relationships that could have appeared to influence the work reported in this paper.
