# Supplementary material for: Regional dopaminergic dysfunction patterns discriminate Parkinson’s disease from multiple system atrophy parkinsonian subtype
Source: Clin Park Relat Disord. 2026 May 23;14:100451. doi: 10.1016/j.prdoa.2026.100451 (PMC13254894; doi:10.1016/j.prdoa.2026.100451)
Supplement: Supplementary Data 1 [file mmc1.docx]

**Supplementary Table 1. Fusion of AAL3 atlas regions into four regions of interest for ^18^F-DOPA PET analysis**

| ROI Names | AAL3 Abbreviation | AAL3 NO. |
| --- | --- | --- |
| Substantia nigra, pars compact | SN_pc_L  SN_pc_R | 161  162 |
| Caudate | Caudate_L  Caudate_R | 75  76 |
| Putamen | Putamen_L  Putamen_R | 77  78 |
| Cerebellum | Cerebelum_Crus1_L  Cerebelum_Crus1_R  Cerebelum_Crus2_L  Cerebelum_Crus2_R  Cerebelum_3_L  Cerebelum_3_R  Cerebelum_4_5_L  Cerebelum_4_5_R  Cerebelum_6_L  Cerebelum_6_R  Cerebelum_7b_L  Cerebelum_7b_R  Cerebelum_8_L  Cerebelum_8_R  Cerebelum_9_L  Cerebelum_9_R  Cerebelum_10_L  Cerebellum_10_R  Vermis_1_2  Vermis_3  Vermis_4_5  Vermis_6  Vermis_7  Vermis_8  Vermis_9  Vermis_10 | 95  96  97  98  99  100  101  102  103  104  105  106  107  108  109  110  111  112  113  114  115  116  117  118  119  120 |

This table details the specific AAL3 atlas regions (Rolls et al., 2015) that were merged to define each ROI used in the voxel‑wise and region‑of‑interest analyses.

Abbreviation: ROI, regions of interest; L, left; R, right; SN_pc, substantia nigra pars compacta. The cerebellar ROI includes all cerebellar lobules and vermian subregions listed above, consistent with the a priori defined mask.
