# Supplementary material for: Regional dopaminergic dysfunction patterns discriminate Parkinson’s disease from multiple system atrophy parkinsonian subtype
Source: Clin Park Relat Disord. 2026 May 23;14:100451. doi: 10.1016/j.prdoa.2026.100451 (PMC13254894; doi:10.1016/j.prdoa.2026.100451)
Supplement: Supplementary Data 2 [file mmc2.docx]

**Supplementary Table 2. Clusters of significantly reduced ^18^F-DOPA uptake in MSA-P compared to PD^*^**

| **Cluster** | **Voxels** | **Peak MNI (x, y, z)** | **Peak intensity (t)** | **Peak region (aal3)** |
| --- | --- | --- | --- | --- |
| 1 | 3976 | 19.5, -76.5, -55.5 | -5.246 | Cerebelum_8_R |
| 2 | 2846 | -30.0, -85.5, -40.5 | -4.531 | Cerebelum_Crus2_L |
| 3 | 619 | 1.5, -39.0, -21.0 | -4.443 | Vermis_1_2 |
| 4 | 801 | -7.5, 3.0, 13.5 | -4.409 | Caudate_L |
| 5 | 755 | 6.0, 13.5, 6.0 | -3.896 | Caudate_R |

*Negative t‑values indicate lower ^18^F-DOPA uptake in MSA‑P than in PD. All clusters survived FDR correction at P<0.05 with an extent threshold of 500 voxels. Abbreviations: MNI, Montreal Neurological Institute.
