# Supplementary material for: Regional dopaminergic dysfunction patterns discriminate Parkinson’s disease from multiple system atrophy parkinsonian subtype
Source: Clin Park Relat Disord. 2026 May 23;14:100451. doi: 10.1016/j.prdoa.2026.100451 (PMC13254894; doi:10.1016/j.prdoa.2026.100451)
Supplement: Supplementary Data 3 [file mmc3.docx]

**Supplementary Table 3. Comparison of regional ^18^F-DOPA SUVR between PD and MSA-P patients (adjusted for age, sex, and disease duration^*^)**

| Region | Group | Estimated marginal mean ± SE | F (1, 26) | P value |
| --- | --- | --- | --- | --- |
| Caudate | PD | 1.610±0.043 | 5.23 | 0.030 |
|  | MSA-P | 1.362±0.056 |  |  |
| Putamen | PD | 1.880±0.099 | 1.33 | 0.259 |
|  | MSA-P | 1.708±0.128 |  |  |
| Cerebellum | PD | 1.058±0.014 | 4.68 | 0.040 |
|  | MSA-P | 1.004±0.018 |  |  |
| SN_pc | PD | 1.404±0.066 | 0.06 | 0.805 |
|  | MSA-P | 1.423±0.085 |  |  |

*Estimated marginal means and statistical comparisons were derived from a univariate general linear model (ANCOVA) with age, sex, and disease duration as covariates. Significant P values (<0.05) are bolded.

Abbreviations: SUVR, standardized uptake value ratio; SE, standard error; SN_pc, substantia nigra pars compacta.
