# Supplementary material for: Regional dopaminergic dysfunction patterns discriminate Parkinson’s disease from multiple system atrophy parkinsonian subtype
Source: Clin Park Relat Disord. 2026 May 23;14:100451. doi: 10.1016/j.prdoa.2026.100451 (PMC13254894; doi:10.1016/j.prdoa.2026.100451)
Supplement: Supplementary Data 4 [file mmc4.docx]

**Supplementary Table 4. ROC analysis of regional SUVR for differentiating MSA‑P from PD**

| Region | AUC (95% CI) | Cut‑off value | Sensitivity (%) | Specificity (%) | Youden index |
| --- | --- | --- | --- | --- | --- |
| Caudate | 0.796 (0.646–0.933) | ≤1.524 | 83.3 | 70.0 | 0.533 |
| Cerebellum | 0.713 (0.538–0.879) | ≤1.021 | 66.7 | 75.0 | 0.417 |

Abbreviation: AUC, area under the curve; CI, confidence interval (bootstrap, 1,000 replicates). The optimal cut‑off was defined as the value maximizing the Youden index. SUVR, standardized uptake value ratio.
