# Supplementary material for: Regional dopaminergic dysfunction patterns discriminate Parkinson’s disease from multiple system atrophy parkinsonian subtype
Source: Clin Park Relat Disord. 2026 May 23;14:100451. doi: 10.1016/j.prdoa.2026.100451 (PMC13254894; doi:10.1016/j.prdoa.2026.100451)
Supplement: Supplementary Data 5 [file mmc5.docx]

**Supplementary Table 5. Correlations between regional ^18^F-DOPA SUVR and clinical metrics**

| **Group** | **Region** | **Clinical metric** | ***r_s_*** | ***P*value** |
| --- | --- | --- | --- | --- |
| PD | Caudate | Score of UPDRS Part III | -0.128 | 0.612 |
|  |  | H-Y stage | -0.203 | 0.392 |
|  |  | Disease duration | -0.377 | 0.101 |
|  | Putamen | Score of UPDRS Part III | -0.381 | 0.119 |
|  |  | H-Y stage | -0.395 | 0.084 |
|  |  | Disease duration | -0.439 | 0.053 |
|  | Cerebellum | Score of UPDRS Part III | 0.257 | 0.303 |
|  |  | H-Y stage | 0.193 | 0.415 |
|  |  | Disease duration | 0.528 | 0.017 |
|  | SN_pc | Score of UPDRS Part III | 0.054 | 0.832 |
|  |  | H-Y stage | -0.144 | 0.545 |
|  |  | Disease duration | -0.186 | 0.432 |
| MSA-P | Caudate | Score of UPDRS Part III | 0.559 | 0.093 |
|  |  | H-Y stage | 0.044 | 0.893 |
|  |  | Disease duration | -0.062 | 0.849 |
|  | Putamen | Score of UPDRS Part III | 0.286 | 0.424 |
|  |  | H-Y stage | 0.306 | 0.334 |
|  |  | Disease duration | 0.015 | 0.964 |
|  | Cerebellum | Score of UPDRS Part III | 0.152 | 0.675 |
|  |  | H-Y stage | 0.480 | 0.114 |
|  |  | Disease duration | 0.007 | 0.982 |
|  | SN_pc | Score of UPDRS Part III | -0.401 | 0.250 |
|  |  | H-Y stage | 0.131 | 0.685 |
|  |  | Disease duration | -0.308 | 0.330 |

Bonferroni-corrected significance threshold: *P*<0.0042*.* Significant correlations are bolded.

Abbreviations: SUVR, standardized uptake value ratio; UPDRS‑III, Unified Parkinson’s Disease Rating Scale Part III; H‑Y, Hoehn‑Yahr; SN-pc, Substantia nigra pars compacta.
